# Functional Monoecy Due to Delayed Anther Dehiscence: A Novel Mechanism in *Pseuduvaria mulgraveana* (Annonaceae)

**DOI:** 10.1371/journal.pone.0059951

**Published:** 2013-03-26

**Authors:** Chun-Chiu Pang, Tanya Scharaschkin, Yvonne C. F. Su, Richard M. K. Saunders

**Affiliations:** 1 School of Biological Sciences, The University of Hong Kong, Hong Kong, China; 2 Earth, Environmental and Biological Sciences, Science and Engineering Faculty, Queensland University of Technology, Brisbane, QLD, Australia; 3 Duke-National University of Singapore Graduate Medical School, Singapore, Singapore; University of Kent, United Kingdom Of America

## Abstract

Unlike most genera in the early-divergent angiosperm family Annonaceae, *Pseuduvaria* exhibits a diversity of floral sex expression. Most species are structurally andromonoecious (or possibly androdioecious), although the hermaphroditic flowers have been inferred to be functionally pistillate, with sterile staminodes. *Pseuduvaria* presents an ideal model for investigating the evolution of floral sex in early-divergent angiosperms, although detailed empirical studies are currently lacking. The phenology and pollination ecology of the Australian endemic species *Pseuduvaria mulgraveana* are studied in detail, including evaluations of floral scent chemistry, pollen viability, and floral visitors. Results showed that the flowers are pollinated by small diurnal nitidulid beetles and are protogynous. Pollen from both hermaphroditic and staminate flowers are shown to be equally viable. The structurally hermaphroditic flowers are nevertheless functionally pistillate as anther dehiscence is delayed until after petal abscission and hence after the departure of pollinators. This mechanism to achieve functional unisexuality of flowers has not previously been reported in angiosperms. It is known that protogyny is widespread amongst early-divergent angiosperms, including the Annonaceae, and is effective in preventing autogamy. Delayed anther dehiscence represents a further elaboration of this, and is effective in preventing geitonogamy since very few sexually mature flowers occur simultaneously in an individual. We highlight the necessity for field-based empirical interpretations of functional floral sex expression prior to evaluations of evolutionary processes.

## Introduction

The Annonaceae is a species-rich, early-divergent angiosperm family consisting of 112 genera and c. 2,440 species [1]. As with most early-divergent angiosperms [2], the majority of Annonaceae species have hermaphroditic, protogynous flowers that are predominantly pollinated by beetles [3]. Unisexual flowers are known to have evolved independently in several disparate lineages within the family [4], *viz. Anonidium* Engl. & Diels [5], *Annona* L. species previously recognized as *Raimondia* Saff. [6], *Diclinanona* Diels [5], *Ephedranthus* S. Moore [7], *Greenwayodendron* Verdc. [8], *Klarobelia* Chatrou [9], *Polyceratocarpus* Engl. & Diels [5], *Pseudephedranthus* Aristeg. [7], *Pseudomalmea* Chatrou [9], *Pseuduvaria* Miq. [10], *Stelechocarpus* (Blume) Hook. f. & Thomson [11], and *Uvariopsis* Engl. [12]. Most of the species in these genera are androdioecious or andromonoecious, and only a few have solely unisexual flowers.

The present study focuses on the genus *Pseuduvaria* (Annonaceae subfam. Malmeoideae tribe Miliuseae [13]), in which 53 of the 57 species have unisexual flowers [10], [14], [15], [16]. Amongst the 41 species that are adequately known, seven (17%) are monoecious (or dioecious), in which all flowers are unisexual, and 31 (76%) are structurally andromonoecious (or androdioecious), in which hermaphroditic and staminate flowers co-occur; in contrast, only two species (5%) possess solely hermaphroditic flowers. These interpretations of floral sex expression, however, are based on assumptions of organ functionality, including the fertility of stamens in hermaphroditic flowers, and the sterility of staminodes in pistillate flowers. These assumptions are based on observations that the staminodes of pistillate flowers are typically smaller than the corresponding stamens in hermaphroditic flowers of the same species, and are often slightly distorted and asymmetrical [10]. The staminodes furthermore often lack pollen, but when pollen is present the grains are sometimes smaller and hence inferred to be sterile: staminode pollen of *Pseuduvaria macrocarpa* (Burck) Y.C.F. Su & R.M.K. Saunders, for example, is c. 20 µm in diameter (equatorial axis), whereas fertile pollen is c. 25 µm [10]. No empirical studies have been conducted to investigate the viability of staminode pollen, however. Floral sex distribution within and among individuals (monoecy vs. dioecy; and andromonoecy vs. androdioecy) is furthermore poorly known for the genus as it cannot easily be inferred from herbarium material.

Most Annonaceae species are beetle-pollinated, and this has been inferred as the plesiomorphic pollination mechanism in the family [3]. *Pseuduvaria*, in contrast, is presumed to be fly-pollinated based on field observations of three species: an unidentified Australian species [17], inferred to be *P. glabrescens* (Jessup) Y.C.F. Su & R.M.K. Saunders based on published photographs and geographical location; *P. froggattii* (F. Muell.) Jessup [18]; and *P. megalopus* (K. Schum.) Y.C.F. Su & Mols [19]. Silberbauer-Gottsberger et al. furthermore suggested that *P. hylandii* Jessup and *P. villosa* Jessup were also probably pollinated by flies [18], but no empirical data was provided. Although several of these species may be sapromyophilous (e.g., *P*. *froggattii* [18], *P*. *megalopus* [19]), it has been suggested that sapromyophily is unlikely to be ubiquitous in the genus as many species (e.g., *P. rugosa* (Blume) Merr. and *P. trimera* (Craib) Y.C.F. Su & R.M.K. Saunders) possess light yellow petals, and lack prominent inner petal glands and dark-colored petal pigmentation [10]. It was suggested that these species attract flies with sweet nectar rather than by mimicking carrion [10].

Our research describes the floral phenology and pollination biology of *Pseuduvaria mulgraveana* Jessup, a narrow endemic from northeastern Queensland, Australia [10]. This species was previously inferred to possess separate staminate and pistillate flowers, with sterile staminodes in the latter [10]. The present study represents the first empirical ecological study of this species and includes the first in vivo and in vitro assessments of the viability of staminode pollen in the family. Results from this study form a sound basis for evaluating sex expression and the evolution of floral unisexuality within the genus, with implications for the Annonaceae as a whole and other early-divergent angiosperms.

## Materials and Methods

### Study site and species

*Pseuduvaria mulgraveana* is a species of small tree (generally < 5 m tall) which bears inflorescences with at most one or two flowers (ca. 15 mm long) [10]. It is geographically very restricted, only occurring in lowland mesophyll vine forests in northeastern Queensland, Australia [10]. Individuals produce relatively few flowers at any one time during the flowering season, unlike some other *Pseuduvaria* species (e.g., *P. froggattii*). *Pseuduvaria mulgraveana* flowers have previously been regarded as unisexual, with structurally hermaphroditic flowers inferred as functionally pistillate with non-viable pollen, although empirical studies are lacking [10]. The seed dispersal mechanism is unknown.

Field observations of *P. mulgraveana* were conducted in the Goldsborough Valley region (17°08'S, 145°45'E) of the Wooroonooran National Park in northeastern Queensland, Australia (permit WITK08297010 from the Environmental Protection Agency, Queensland Government, issued to T. Scharaschkin). This area is mainly composed of lowland rainforest, c. 40 m asl, dominated by Apocynaceae, Araliaceae, Euphorbiaceae, Lauraceae, Moraceae, Myrtaceae, and Sapindaceae [20].

A population of *P. mulgraveana* (17°14'20" S, 145°46'54" E) was selected for study within Goldsborough Valley, and 19 flowering individuals labelled with plastic tags for identification in phenological and experimental studies. Voucher specimens from this population are held in BRI herbarium (e.g., *G. Sankowsky & N. Sankowsky 336*).

### Floral phenology

A flower-level phenological study of *P. mulgraveana* was conducted over a one-month period (October–November 2010). Phenological changes in the flowers were monitored by tagging a total of 30 flower buds from 19 individual trees. All flowers encountered were used for the phenological observation irrespective of their positions on the plants. Observations on floral morphology and sexual functioning were taken at 1-h intervals from initial petal opening until the end of anthesis. The pistillate phase was recognized by the presence of stigmatic exudate (which has previously been shown to be correlated with stigmatic receptivity: e.g. [21], [22]), whilst the onset of the staminate phase was determined by anther dehiscence. Other morphological and/or phenological changes of the flowers were also recorded, including scent emission, petal color, and petal orientation.

A phenological study at the individual tree level, including an assessment of possible flowering synchrony (or synchronous dichogamy [23], [24]), was undertaken by surveying the number of flowers in pistillate and staminate phases from 10 plants over 10 consecutive days.

### Floral thermogenesis and scent analysis

A hand-held thermometer (accuracy ± 0.1°C) was used to record the temperature inside the floral chambers of 10 flowers from 10 different individuals during different phenological stages, with measurements taken at 1-h intervals. Ambient temperature immediately outside the floral chamber was recorded simultaneously.

Volatile floral scent compounds were sampled using a purified SPME (solid-phase micro-extraction) fiber with a 65-µm divinylbenzene/polydimethylsiloxane coating, mounted on a manual sampling device (Supelco, Bellefonte, PA). Flowers at different developmental stages (including pre-receptive, pistillate-phase, staminate-phase, and post-receptive flowers) were collected and placed in polypropylene bags [25], which were immediately sealed to limit air movement. Flowers were randomly selected amongst 11 individuals, with two replicate samples for pre-receptive phase flowers, and three replicates for each of the sexually mature phases. The SPME fiber was then inserted into the bag and exposed for 2 h to enable adsorption of volatile compounds.

The fibers were subsequently transferred to the School of Biological Sciences, The University of Hong Kong, for gas chromatography-mass spectrometry (GCMS) analysis using an Agilent 6890N gas chromatograph (Agilent Technologies, Palo Alto, CA) coupled to an Agilent 5973 mass selective detector with a 30 m×0.255 mm i.d. DB-WAX capillary column and a 0.25 µm film (J & W Scientific, Folsom, CA). Helium was used as a carrier gas, with an injection temperature of 250°C for 1 min to allow vaporization of volatile compounds. The oven was maintained at 50°C for the first 5 min and then raised by 5°C/min to 230°C, and then at 230°C for 20 min. Electron ionization mass spectrometry was used with an acquisition range of 30–650 m/z. The volatile compounds were identified by comparing their mass spectra with the NIST 02 MS library bundle (National Institute of Standards & Technology, Gaithersburg, MD). Compounds with estimated identity likelihoods below 80 percent were considered as unknown [26]. Kovats index values were calculated against *n*-alkane standards to confirm compound identity [27]. Smaller alkanes with retention times less than 5 min were not comparable to others, and hence Kovats index values were not calculated.

### Pollen viability

Pollen grains were obtained from both staminate flowers (staminate phase) and hermaphroditic flowers (pistillate, petal abscission, and staminate phases). In vitro pollen germination tests were used to evaluate the optimal sucrose concentration for pollen germination and also to assess pollen viability. Artificial sucrose solutions of different concentrations (0%, 5%, 10%, 15%, 20%, 25% and 30% by weight) were mixed with 50% H_3_BO_3_ and 50% Ca(NO_3_)_2_ by volume [28]. Freshly harvested pollen grains (obtained randomly from 19 individuals) were mixed with each sucrose solution on glass slides; the slides were maintained in closed Petri dishes at ambient temperature for 24 h, and then observed under a light microscope to determine the proportion of germinated pollen grains (using six replicates for each sucrose concentration). The statistical significance of germination rates between different pollen sources at the optimal sucrose concentration was assessed by analysis of variance after checking normality and testing for equal variance and discriminated using the Tukey test for unequal *n*.

### Floral visitors

Observations of floral visitors to *P. mulgraveana* (21 flowers from 11 individuals) were initiated as soon as flowers became sexually mature. Observations were made at 2-h intervals in order to monitor arrival/departure times of floral visitors and their activities during each visit. The following criteria were specifically adopted to determine whether the floral visitors qualify as effective pollinators: (1) the presence of *P. mulgraveana* pollen on the body of the floral visitor, assessed using a light microscope; (2) the coincidence of floral visits with the receptive periods of the flower; (3) relative visitation rates; and (4) evidence of inter-floral movement of the floral visitors.

Samples of floral visitors (including 52 beetles from 11 flowers) were immobilized in Eppendorf tubes containing absorbent paper soaked with chloroform. After checking for the presence of pollen grain attachment, the insects were stored in 70% industrial methylated spirits for expert identification (by Dr Carl Wardhaugh, University of Melbourne).

## Results

### Floral phenology

**Figure 1:**
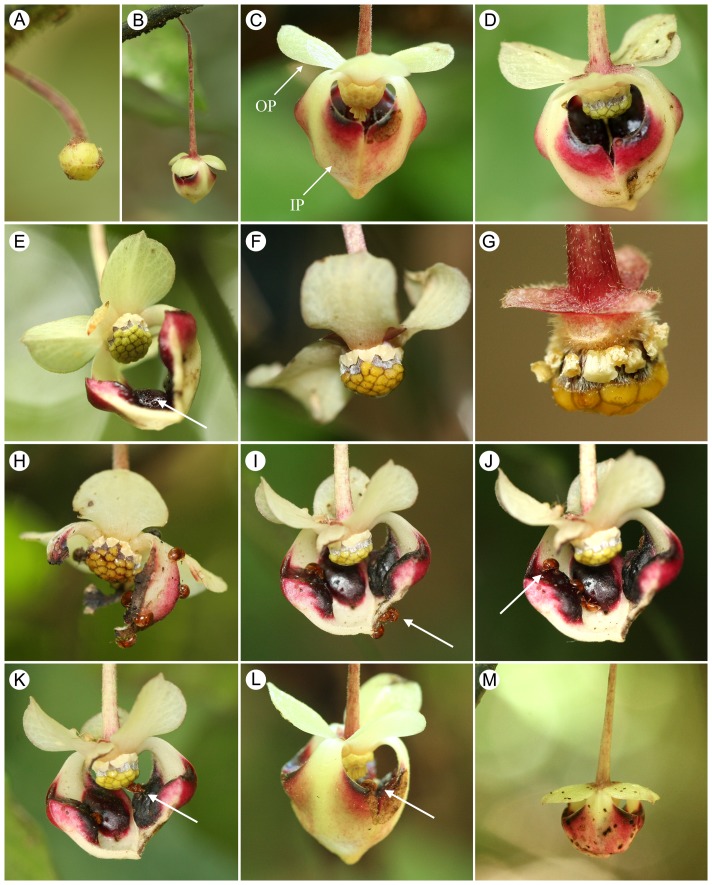
Flowers of *Pseuduvaria mulgraveana*, showing different phenological stages (A–G) and activities of the effective beetle pollinator, *Aethina australis* (H–M). (A) Early bud phase. (B) Outer petal opening phase. (C) Sexually mature staminate flower, with innermost anthers dehiscing (OP  =  outer petal; IP  =  inner petal). (D) Hermaphroditic flower in pistillate phase. (E) Hermaphroditic flower during pistillate phase, with nectar secretion (indicated by arrow) on the dark red glands. (F) Hermaphroditic flower in petal abscission phase. (G) Hermaphroditic flower in staminate phase, with anthers dehiscing. (H) Beetles consuming petals and nectar in a pistillate-phase hermaphroditic flower. (I) Beetles copulating (indicated by arrow) outside the floral chamber of a pistillate-phase hermaphroditic flower. (J) Beetles remaining on the inner petal glands of a pistillate-phase hermaphroditic flower (indicated by arrow). (K) Beetles touching stigmas of a pistillate-phase hermaphroditic flower (indicated by arrow). (L) Beetles (indicated by arrow) visiting sexually mature staminate flowers. (M) Beetle bite marks on the petals.

**Figure 2:**
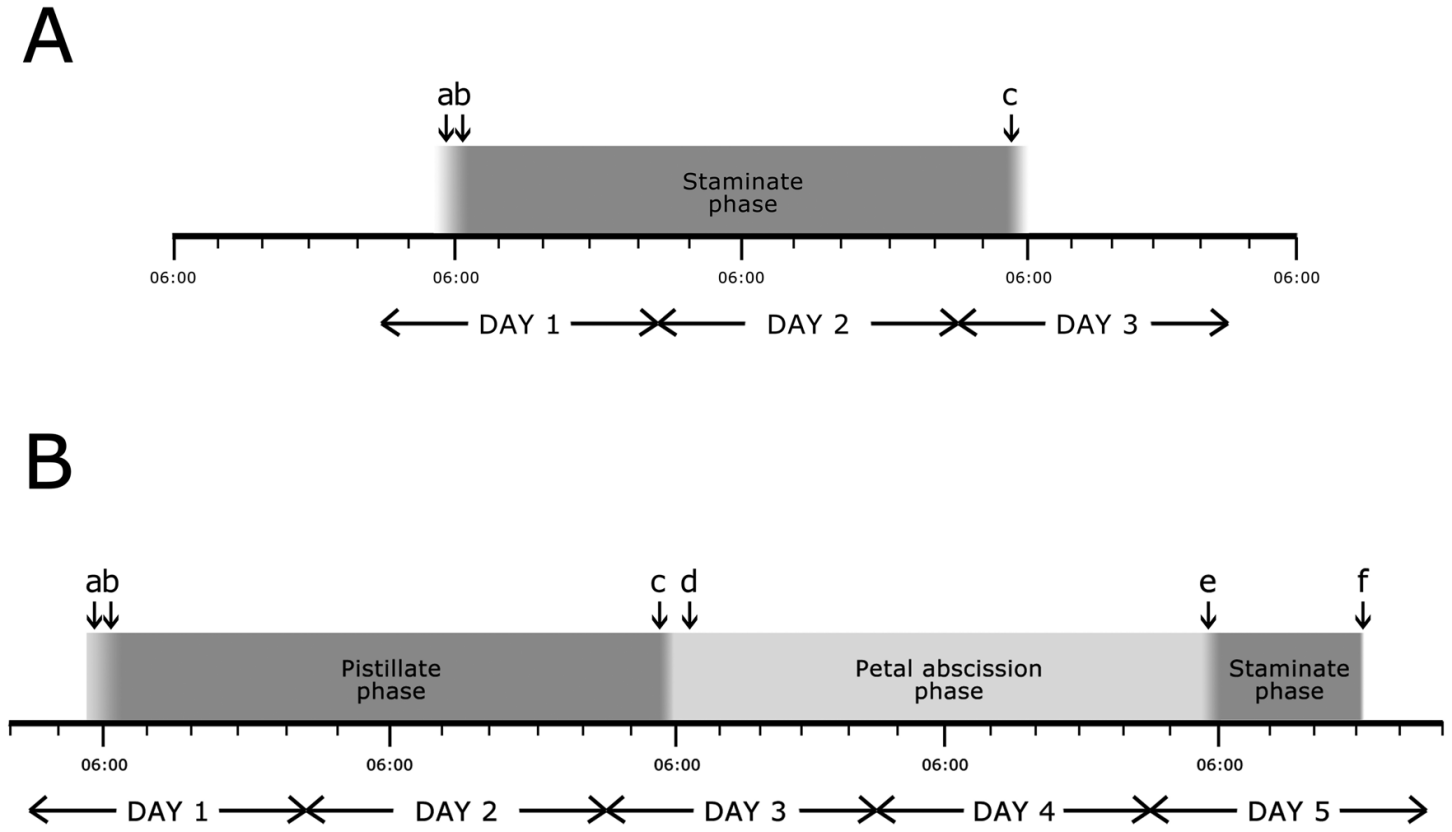
Timing of phenological events in flowers of *Pseuduvaria mulgraveana* during sexually functional phases. (A) Staminate flowers: a  =  initiation of anther dehiscence and nectar secretion; b  =  arrival of pollinators; c  =  petal abscission and departure of pollinators. (B) Structurally hermaphroditic flowers: a  =  start of stigma exudation and nectar secretion; b  =  arrival of pollinators; c  =  petal abscission and departure of pollinators; d  =  cessation of stigmatic receptivity; e  =  initiation of anther dehiscence of the staminodes; f  =  abscission of staminodes. Numbering of days in (A) and (B) is not correlated.

Two different floral morphs are evident in populations of *Pseuduvaria mulgraveana*, reflecting differences in sexual expression (Fig. 1): structurally hermaphroditic flowers with both carpels and stamens; and staminate flowers that lack carpels. These two floral morphs inevitably display differing phenological patterns (Fig. 2). The average duration of staminate and hermaphroditic flowers from the early bud stage to the end of the staminate phase is c. 19 and 23 d, respectively. The durations of the staminate phase in staminate flowers and pistillate phase in hermaphroditic flowers are both c. 2 d, although in the latter, the pistillate phase is followed by the petal abscission phase of variable duration (2–5 d) and then a short (c. 12 h) staminate phase.

The staminate flowers show three distinct phases (Fig. 2A), as follows:

- Early bud phase (c. 15 d; Fig. 1A): The flower buds are initially slightly conical (subsequently becoming globose) and are partially enclosed by the calyx. The outer whorl of petals is visible from bud inception, whereas the inner petals are only exposed a week later, as the bud enlarges. The calyx and corolla are pale green throughout this stage. The pedicel elongates as the flower bud grows.
- Outer petal opening phase (c. 2 d; Fig. 1B): The outer petals begin to reflex, forming three lateral apertures between the claws of the inner petals, whilst the inner petals remain apically coherent, forming a partially enclosed mitriform floral chamber that persists throughout the lifespan of the flower. Although the reproductive organs are visible, they are immature at this stage. The inner petals develop a pinkish-purple pigmentation around the margins of the floral apertures. The flower bud achieves its maximum size by the end of this stage.
- Staminate phase (c. 2 d, from c. 0500 h on day 1 until c. 0500 h on day 3; Fig. 1C): The outer petals further separate and become strongly reflexed. The onset of this phase is indicated by the dehiscence of the anthers (a in Fig. 2A). The androecium matures centrifugally, with stamens at the center of the androecium maturing before those at the margin. The stamens subsequently abscise from the receptacle and collect in the inverted mitriform chamber formed by the inner petals, together with previously released pollen. Nectar is secreted from the inner petal glands and is mixed with the released pollen grains and abscised stamens. No floral odour is detectable to human perception. Pollinators are attracted to the flower from the beginning of this stage (b in Fig. 2A) and access the chamber through the lateral apertures; they are active inside of the floral chamber and consume nectar and pollen, inadvertently collecting pollen. The mitriform chamber abscises towards the end of this stage (c in Fig. 2A), dispersing the aggregated stamens and encouraging the departure of pollinators; this stage resembles the petal abscission phase described below for hermaphroditic flowers.The structurally hermaphroditic flowers show five different phases (Fig. 2B), of which the first two (‘early bud’ and ‘outer petal opening’ phases) are similar to those of staminate flowers, as described above. The following three phases occur after the separation of the outer petals:
- Pistillate phase (c. 2 d, from c. 0500 h on day 1 until c. 0500 h on day 3; Fig. 1D): The outer petals become strongly reflexed. The onset of receptivity in the early morning (a in Fig. 2B) is indicated by the formation of a thin layer of exudate on the stigmas; the anthers remain intact throughout this phase. Nectar is secreted from the inner petal glands (Fig. 1E; a in Fig. 2B). No odor is detectable to human perception. The onset of stigmatic receptivity coincides with the arrival of pollinators (b in Fig. 2B), which gain access to the floral chamber through the apertures between the inner petals. The pollinators are active inside the chamber and were observed to make physical contact with the stigmas.
- Petal abscission phase (c. 2–5 d; Fig. 1F): The mitriform inner petal whorl abscises as a whole (c in Fig. 2B), followed by the outer petals, which detach separately, encouraging the pollinators to leave the flower. The stigmatic receptivity of the flowers decreases during this stage, with cessation of stigmatic exudate formation and consequent drying of stigmas (d in Fig. 2B). No odor is detectable to human perception. The anthers are not dehiscent, indicating that autogamy is unlikely as there is no temporal overlap between pollen presentation and stigmatic receptivity, regardless of the viability of the staminode pollen. The duration of the petal abscission phase is not strictly synchronized and varies amongst flowers, which is presumably governed by the timing of the subsequent phase.
- Staminate phase (c. 12 h, from c. 0500 h until c. 1700 h on the same day; Fig. 1G): The stamens/staminodes separate slightly from the gynoecium and the anthers dehisce (e in Fig. 2B), although pollen release is not as obvious as in staminate flowers because there are fewer stamens/staminodes and each has less pollen. By the end of this stage, almost all stamens/staminodes have abscised from the flower (f in Fig. 2B). This stage is sometimes completely omitted, so that the entire flower, including the carpels and torus, abscises before anther dehiscence. Pollinators do not visit the flowers during the staminate phase since the mitriform dome-like chamber, presumably the major attractant, is lacking.

Both staminate and hermaphroditic flowers co-occur within a single individual, and *P. mulgraveana* is consequently regarded as andromonoecious. There is no evidence of the presence of flowering synchrony in *P. mulgraveana*; geitonogamy is therefore feasible, although there are very few sexually mature flowers co-occurring in an individual at any one time.

### Floral thermogenesis and scent analysis

There is no evidence of floral heat production during the sexually functional phases of either floral morph. Neither hermaphroditic nor staminate flowers of *P. mulgraveana* emit any fragrance during the sexually functional phases that is detectable to humans.

GCMS enabled the identification of a combined total of 106 volatile compounds from the scents of hermaphroditic and staminate flowers during different phenological stages (Table S1). The majority of volatile compounds are emitted by immature flowers (stage II): 86 compounds (81%) occur in immature hermaphroditic flowers, and 60 (57%) occur in immature staminate flowers. No floral visitors or pollinators visit immature flowers so that these compounds presumably do not serve as attractants. In hermaphroditic flowers, the following eight compounds were found (> 0.25% of total peak area) exclusively in pistillate-phase flowers and not immature flowers: (Z)-3,7-dimethyl-1,3,6-octatriene; 1,3-dichlorobenzene; 1,3,3-trimethyl-2-oxabicyclo[2.2.2]octan-6-ol; 4-methyltetradecane; thymol methylether; 2-cyclopropyl-2-methyl-N-(1-cyclopropylethyl)-cyclopropane carboxamide; and two unidentified compounds. In staminate flowers, the following six compounds were found (> 0.25% of total peak area) exclusively in staminate-phase flowers when compared with immature flowers: diethyltoluamide; 1,4,7,10,13,16-hexaoxacyclooctadecane; caryophyllene; azulene; and two unidentified compounds. 2,2'-oxybis-ethanol forms one of the main components of the floral scent of *P. mulgraveana*, with an increase from 6.3% and 5.4% of total peak area in immature hermaphroditic and staminate flowers, respectively, to 14.7% in pistillate-phase flowers and 16.9% in staminate-phase flowers (Table S1).

### Pollen viability

**Figure 3:**
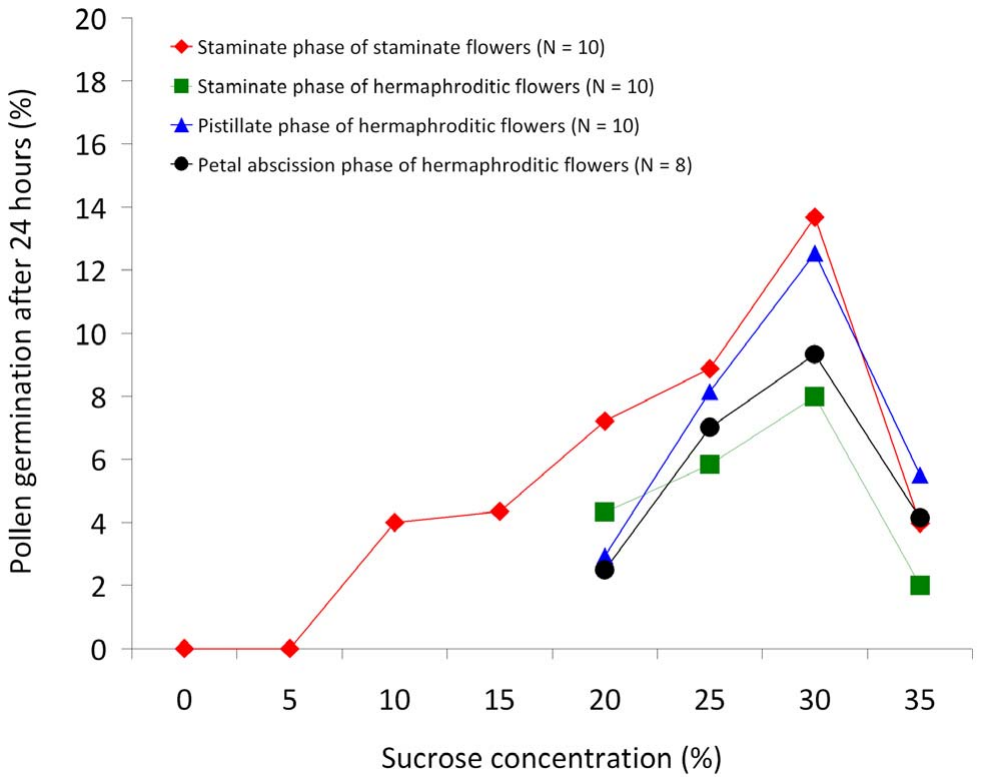
Comparative germination of *Pseuduvaria mulgraveana* pollen across eight different sucrose concentrations. Pollen obtained from different phenological phases (pistillate, petal abscission, and staminate phases) of staminate and structurally hermaphroditic flowers.

The highest level of pollen germination was found in 30% sucrose solution (Fig. 3). Staminate and hermaphroditic flowers both produce viable pollen during the staminate phase, with 13.7% and 8.0% pollen germination from the two floral morphs, respectively. Intriguingly, 12.5% and 9.3% of pollen from the pistillate phase and petal abscission phase of hermaphroditic flowers also germinates in 30% sucrose solution, respectively, suggesting that pollen is fully developed prior to anther dehiscence. There is no significant difference in the levels of pollen germination irrespective of whether the pollen is produced in staminate or hermaphroditic flowers (one-way ANOVA with arcsine transformation, df  =  3, *F*  =  0.370, *P*  =  0.775).

### Floral visitors

Two species of insect were observed visiting the flowers of *P. mulgraveana*: a small nitidulid beetle, *Aethina australis* Kirejtshuk (Fig. 1H–L; entire body c. 2 mm long, abdomen c. 1 mm wide), and an unidentified fly. *Aethina australis* was attracted to the flowers during the early morning (around 0600 h), and generally stayed inside the chambers throughout the sexually functional phases until the floral chamber abscised. In hermaphroditic flowers, no visitors were observed during the petal abscission phase or staminate phase. The beetles are active on the adaxial surface of the chamber, brushing against the stigmas and stamens and potentially pollinating the flowers or increasing their pollen load. Copulation of the beetles was observed both inside and outside the floral chamber (Fig. 1I). Bite marks and areas of petal consumption were furthermore observed in the flowers (Fig. 1M).

The unidentified fly species was also observed entering the floral chamber, although they were relatively rare visitors compared to *A. australis*, with only six individuals observed during the entire three-week survey. The flies visited both pistillate-phase hermaphroditic flowers and staminate-phase staminate flowers, but pollen grains were not observed on their bodies.

## Discussion

### Determination of effective pollinator

The beetle *Aethina australis* (Coleoptera: Nitidulidae) is inferred as an effective pollinator of *Pseuduvaria mulgraveana* based on the following criteria: the beetles were observed to be abundant on *P. mulgraveana* flowers, with their arrival and departure times coincident with the sexually functional phases of the flowers; and pollen of *P. mulgraveana* was observed on their bodies, including those collected from pistillate-phase hermaphroditic flowers, providing unequivocal evidence of inter-floral movement of beetles. *Aethina* Erichson species have previously been identified as pollinators of other Annonaceae species, including *Uvaria semecarpifolia* Hook. f. & Thomson, pollinated by *Aethina* (*Circopes*) *subquadrata* Motschulsky [29].

Previous studies of the pollination biology of *Pseuduvaria* species have indicated fly pollination [17], [18], [19]. The possibility that the unidentified fly species observed visiting flowers of *P. mulgraveana* may act as a pollinator cannot be precluded; this would certainly be consistent with morphological characteristics of the flowers that are indicative of fly pollination, including the localized areas of dark red-purple pigmentation on the petals, and the presence of nectary glands. The observation of beetle pollination in flowers with adaptations indicative of myophily is also observed in *Mitrephora* (Blume) Hook. f. & Thomson, which shares a similar floral morphology to *Pseuduvaria*, with inner petals forming a small, partially enclosed mitriform chamber [30]: although van der Pijl suggested on the basis of floral morphology that *Mitrephora* is likely to be pollinated by flies [31], empirical data demonstrates that at least one species, *M. heyneana* (Hook. f. & Thomson) Thwaites, is pollinated by small nitidulid beetles [30].

### Pollinator attractants and rewards

The absence of a noticeable floral scent in *P. mulgreaveana* detectable by human olfaction contrasts with reports for other *Pseuduvaria* species such as *P. froggattii*, which apparently emits a floral scent reminiscent of ‘old dishwater and vomit’ [18], and an unidentified *Pseuduvaria* species (probably *P. glabrescens*), with a ‘slightly perfumed’ fragrance [17]. Despite the apparent absence of a floral scent in *P. mulgraveana*, GCMS analysis of odors from sexually mature flowers reveal a mixture of volatile compounds previously implicated as insect attractants and/or pheromones. Of the eight volatiles identified from pistillate-phase hermaphroditic flowers (but absent from immature flowers), (Z)-3,7-dimethyl-1,3,6-octatriene and thymol methylether have been reported to be attractants and/or pheromones for Coleoptera and other orders of insects [32]. Similarly, of the six volatiles identified from sexually mature staminate flowers (but absent from immature flowers), caryophyllene is known to be an attractant for Hymenoptera [32], although this has not been reported for Coleoptera. Caryophyllene is commonly reported in the Annonaceae, occurring in *Anaxagorea brevipes* Benth., *Anaxagorea dolichocarpa* Sprague & Sandwith, *Annona neoinsignis* H. Rainer, *Desmos chinensis* Lour., *Duguetia asterotricha* (Diels) R.E. Fr., *Uvaria cordata* (Dunal) Alston, *Uvaria semecarpifolia* Hook. f. & Thomson, *Xylopia aromatica* (Lam.) Mart., and *Xylopia benthamii* R.E. Fr. [29], [33], [34].

Although nectar production in *Pseuduvaria* was previously interpreted as an adaptation to attract flies [18], the nectar produced by the inner petal glands in *P. mulgraveana* clearly functions as a nutritive reward to *A. australis*, as the beetles were observed consuming it. Beetle- and fly-pollinated flowers often share similar traits, possibly explaining the attraction of both beetles and flies to the same flowers. Differentiating between fly-pollinated and beetle-pollinated Annonaceae species is often difficult, a problem also encountered in other fly- and beetle-pollinated families such as the Araceae [35], [36]. Saunders has hypothesized that evolutionary shifts between beetle and fly pollination in the Annonaceae are unlikely to have been significantly constrained due to the similarities between the adaptations to these different pollinator guilds [3]. Additional empirical observations are necessary in order to determine possible evolutionary transitions in pollination system in *Pseuduvaria*, especially for species in early-divergent clades with pale-colored petals and lacking nectaries, such as *P. galeata* J. Sinclair, *P. rugosa* and *P. trimera* [14].

Petal consumption as a form of nutritive reward is particularly common in large scarab-beetle pollination, and has been reported in various Annonaceae genera, including *Annona* L., *Cymbopetalum* Benth. and *Duguetia* A. St.-Hil. [18], [37], [38], [39], [40]. This nutritive reward is also evident for small beetles, since *P. mulgraveana* petals were observed to be eaten by *A. australis* (Fig. 1H). As well as large areas of petal consumption, small bite marks were also observed; this observation, together with the fact that the beetles copulate in the flowers, suggests that the petals may also serve as potential oviposition sites for the beetles (see also [41]) although further investigation into the use of the petals is required.

### Structural and functional interpretations of floral sex

Empirical data reveal that *P. mulgraveana* is andromonoecious (i.e., staminate and hermaphroditic flowers co-occur within a single individual plant), with no evidence of inter- and intra-individual flowering synchrony. The stamens in hermaphroditic flowers were shown to be fertile, despite differences in shape and size from those in staminate flowers. The possible selective advantage of andromonoecy in *P. mulgraveana* may be explained by considering the phenological timing of sexual maturation. The hermaphroditic flowers of *P. mulgraveana* are unusual in showing abscission of the corolla prior to anther dehiscence. This delayed anther dehiscence (or premature petal abscission) is likely to have a profound impact on the functioning of the flower, since the floral chamber is the source of many of the attractants and rewards offered to the pollinators, including visual and olfactory cues, nutritive rewards (both as petal tissue and nectar from the inner petal glands), and possibly also as a sheltering and/or tryst site. Abscission of the corolla inevitably results in departure of the pollinators prior to pollen release, and hence precludes transfer of pollen from hermaphroditic flowers. The hermaphroditic flowers are therefore functionally pistillate, and the structural andromonoecy of *P. mulgraveana* populations actually functions as true monoecy. This mechanism to achieve functional unisexuality of flowers has not been reported previously in the Annonaceae (or apparently amongst angiosperms as a whole), and has implications for inferences regarding the evolution of unisexual flowers. Although structurally hermaphroditic (but functionally pistillate) flowers co-occur with staminate flowers in *P. mulgraveana*, this functional monoecy is likely to effectively promote xenogamy: individual trees typically bear relatively few sexually mature flowers simultaneously, and the opportunities for intra-individual pollen transfer are therefore reduced, limiting the possibility of geitonogamy.

Difficulties are encountered in determinations of floral sex expression based solely on the examination of herbarium specimens. Staminate and hermaphroditic flowers may not always be present in the same branch in andromonoecious species, for example, leading to the erroneous interpretation of androdioecy. This problem is known to occur in *Pseuduvaria*: *P. trimera*, for example, has been observed to bear staminate and structurally hermaphroditic flowers on separate branches within the same individual (C.-C. Pang, pers. obs.). Field-based observations are therefore essential for determining floral sex expression in such taxa.

### Evolutionary pathways to floral unisexuality

Floral hermaphroditism is widespread in the Annonaceae, occurring in all major lineages including the basalmost genus *Anaxagorea* [42], [43] and the Late Cretaceous fossil *Futabanthus asamigawensis* Takahashi et al., which is hypothesized to have a phylogenetic position at the crown of the Annonaceae clade [44]. Despite the lack of adequate resolution in recent molecular phylogenetic studies of subfamily Malmeoideae and the resulting uncertainty in the sister-group relationship of *Pseuduvaria* (e.g., [15]), it is clear that *Pseuduvaria* species with unisexual flowers have evolved from ancestors with hermaphroditic flowers.

Su et al. developed hypotheses to explain the evolution of floral sex expression in *Pseuduvaria* based largely on assumptions that structurally hermaphroditic flowers possess sterile staminodes and that any pollen formed is likely to be non-viable [14]. The authors used parsimony character optimization to conclude that unisexual flowers were plesiomorphic in the genus, with evidence of at least seven independent evolutionary reversals to the hermaphroditic condition in disparate lineages [14]. The evidence presented here on the formation of fertile pollen in structurally hermaphroditic flowers of *P. mulgraveana*, however, indicates the need for a reappraisal of the assumptions underlying this previous analysis.

Most *Pseuduvaria* species have inflorescences that consist of relatively few flowers, although some have up to 15; even in multi-flowered inflorescences, however, only one flower typically matures at any one time within an inflorescence [10]. The inflorescences are generally solitary or paired in *Pseuduvaria*, but occur in clusters of up to 30 in some species, increasing the likelihood of multiple flowers being receptive simultaneously and the potential for geitonogamous pollination. Many species in clade IV (sensu [15]) bear large inflorescence clusters and are therefore likely to bear multiple receptive flowers simultaneously, including the monoecious/dioecious species *P. latifolia* (Blume) Bakh. f., *P. mindorensis* Y.C.F. Su & R.M.K. Saunders and *P. parvipetala* Y.C.F. Su & R.M.K. Saunders [10]. Similar congruence of data is not apparent for the species in clade III, however, as *P. fragrans* Y.C.F. Su et al. and *P. phuyensis* (R.M.K. Saunders et al.) Y.C.F. Su & R.M.K. Saunders, which are monoecious or dioecious, only bear solitary or paired inflorescences [15], [45]. The formation of multiple flowers on an individual plant that are simultaneously receptive is likely to increase potential reproductive success in dioecious species; significantly, Silberbauer-Gottsberger et al. reported that *Pseuduvaria froggattii* (clade V), which produces large clusters of inflorescences, is possibly dioecious [18]. An individual of *P. froggattii* was observed by the first author in Atherton, Queensland, to produce only hermaphroditic flowers, however, suggesting that androdioecy cannot be satisfactorily ruled out (although further study is required to investigate if it is functionally dioecious).

Empirical data demonstrating the existence of andromonoecy in *P. mulgraveana* and dioecy/androdioecy in *P. froggattii* clearly show that these two (or three) conditions coexist in the genus. The evolutionary relationship between andromonoecy and androdioecy is obscure. Lloyd proposed that dioecy may be derived from monoecy through the ‘paradioecy pathway’ [46], in which a gradual divergence in sex allocation occurs within a population, with some individuals producing more staminate flowers, and the others producing more pistillate flowers. There are no corresponding studies of androdioecious/andromonoecious species, however, and it is suggested that *Pseuduvaria* could be a potential candidate genus for such investigations.

## Conclusions

This study documents the floral phenology and pollination biology of *P. mulgraveana* in its natural range in northeastern Queensland, Australia. Empirical studies and field surveys reveal that it is andromonoecious, with hermaphroditic and staminate flowers co-occurring within individuals. Both floral morphs were shown to produce viable pollen grains, contradicting the prevailing hypothesis that the stamens of structurally hermaphroditic flowers are sterile. A novel mechanism to promote xenogamy is reported. The structurally hermaphroditic flowers of *P. mulgraveana* exhibit delayed anther dehisce, with pollen only released after petal abscission once the pollinators have departed. Although structurally hermaphroditic, these flowers are therefore functionally pistillate, and effectively promote xenogamy by precluding autogamy and limiting geitonogamy. In contrast with previous studies of other species in the genus, *P. mulgraveana* was shown to be beetle-pollinated, with petal tissue and nectar provided as nutritive rewards to the pollinators.

## Supporting Information

Table S1 Putative chemical composition of floral volatiles emitted by hermaphroditic and staminate flowers in *Pseuduvaria mulgraveana*. Phenological phases: II  =  immature; III  =  pistillate phase; IV  =  petal abscission phase; V  =  staminate phase.
(DOC)
