# Supplementary material for: Functional Monoecy Due to Delayed Anther Dehiscence: A Novel Mechanism in Pseuduvaria mulgraveana (Annonaceae)
Source: PLoS One. 2013 Mar 26;8(3):e59951. doi: 10.1371/journal.pone.0059951 (PMC3608580; doi:10.1371/journal.pone.0059951)
Supplement: Table S1 — Putative chemical composition of floral volatiles emitted by hermaphroditic and staminate flowers in Pseuduvaria mulgraveana. Phenological phases: II = immature; III = pistillate phase; IV = petal abscission phase; V = staminate phase. (DOC) [file pone.0059951.s001.doc]

TABLE S1. Putative chemical composition of floral volatiles emitted by hermaphroditic and staminate flowers in *Pseuduvaria mulgraveana*. Phenological phases: II = immature; III = pistillate phase; IV = petal abscission phase; V = staminate phase.

|  |  |  |  | Identity likelihood | | | | | Percentage of total peak area | | | | |
| --- | --- | --- | --- | --- | --- | --- | --- | --- | --- | --- | --- | --- | --- |
| No. | Compound name ^a^ | Mean RT (min) | Kovats Index | Hermaphroditic flowers | | | Staminate flowers | | Hermaphroditic flowers | | | Staminate flowers | |
|  |  |  |  | II | III | IV | II | V | II | III | IV | II | V |
| 1 | Cyclopentasiloxane, decamethyl- | 6.52 | 1255 | 91 | 95 | 94 | 95 | 95 | 0.57 | 1.53 | 0.87 | 1.79 | 1.00 |
| 2 | (Z)-3,7-Dimethyl-1,3,6-octatriene | 7.14 | 1293 | — | 96 | — | — | — | 0.00 | 0.26 | 0.00 | 0.00 | 0.00 |
| 3 | Dodecane | 7.98 | 1340 | na | 91 | 90 | 91 | 87 | 0.54 | 3.04 | 1.84 | 4.89 | 3.23 |
| 4 | Eicosane | 8.26 | 1355 | 93 | 91 | — | na | 80 | 1.05 | 1.80 | 0.00 | 3.79 | 1.14 |
| 5 | Tetradecane, 4-methyl- | 8.65 | 1374 | — | 86 | na | 86 | 91 | 0.00 | 1.01 | 0.14 | 1.02 | 0.92 |
| 6 | Tridecanol, 2-ethyl-2-methyl- | 8.75 | 1379 | — | — | — | 90 | 90 | 0.00 | 0.00 | 0.00 | 0.90 | 0.23 |
| 7 | Pentadecane | 9.69 | 1422 | 80 | 91 | 94 | 91 | 91 | 3.81 | 5.29 | 4.67 | 4.72 | 4.44 |
| 8 | Unknown | 10.15 | 1442 | na | na | na | na | na | 0.38 | 0.79 | 0.89 | 0.78 | 0.84 |
| 9 | Unknown | 10.20 | 1444 | — | na | na | — | na | 0.00 | 1.64 | 2.00 | 0.00 | 0.93 |
| 10 | Unknown | 10.22 | 1445 | na | na | — | na | na | 1.38 | 1.98 | 0.00 | 3.12 | 2.00 |
| 11 | Tetratriacontane | 10.64 | 1462 | 83 | 87 | na | 87 | 90 | 0.40 | 0.57 | 0.15 | 0.83 | 0.59 |
| 12 | Cyclohexasiloxane, dodecamethyl- | 12.66 | 1535 | 87 | 93 | na | 91 | 90 | 0.66 | 1.02 | 0.92 | 1.25 | 0.90 |
| 13 | Benzene, 1,4-dichloro- | 13.31 | 1556 | 96 | 97 | 96 | 97 | 97 | 0.51 | 0.30 | 0.90 | 1.56 | 0.44 |
| 14 | Benzene, 1,3-dichloro- | 13.36 | 1558 | — | 96 | — | — | 86 | 0.00 | 0.43 | 0.00 | 0.00 | 0.16 |
| 15 | Octacosane | 14.36 | 1588 | 87 | 87 | 86 | 86 | 87 | 1.41 | 0.97 | 0.56 | 1.45 | 1.29 |
| 16 | Copaene | 14.45 | 1591 | na | 98 | 98 | 97 | 98 | 0.29 | 0.73 | 2.17 | 1.58 | 0.82 |
| 17 | Unknown | 15.27 | 1614 | na | na | na | na | na | 1.83 | 0.59 | 1.43 | 0.65 | 1.22 |
| 18 | Unknown | 15.35 | 1617 | — | na | — | na | na | 0.00 | 0.94 | 0.00 | 1.10 | 0.17 |
| 19 | Unknown | 15.45 | 1619 | na | na | — | — | na | 0.56 | 0.11 | 0.00 | 0.00 | 0.18 |
| 20 | Heptadecane, 8-methyl- | 15.62 | 1624 | na | 86 | na | na | 90 | 1.87 | 1.34 | 0.79 | 1.24 | 1.11 |
| 21 | Unknown | 15.89 | 1631 | na | na | na | na | na | 0.82 | 0.78 | 0.64 | 0.93 | 0.56 |
| 22 | Unknown | 16.00 | 1634 | na | na | na | na | na | 0.47 | 0.05 | 0.23 | 0.27 | 0.14 |
| 23 | Unknown | 16.60 | 1650 | na | na | na | na | na | 1.58 | 2.78 | 2.78 | 3.82 | 1.82 |
| 24 | Unknown | 16.72 | 1653 | na | na | na | na | na | 0.31 | 0.66 | 0.32 | 0.52 | 0.29 |
| 25 | Caryophyllene | 17.09 | 1662 | na | — | 96 | — | 90 | 0.37 | 0.00 | 0.54 | 0.00 | 0.31 |
| 26 | Cyclopropane carboxamide, 2-cyclopropyl-2-methyl-N-(1-cyclopropylethyl)- | 17.16 | 1664 | — | 86 | — | na | — | 0.00 | 0.29 | 0.00 | 0.33 | 0.00 |
| 27 | Unknown | 18.05 | 1685 | na | na | na | na | na | 0.25 | 0.65 | 0.65 | 1.02 | 0.39 |
| 28 | Unknown | 18.19 | 1688 | na | na | na | na | na | 0.15 | 0.52 | 0.49 | 0.83 | 0.37 |
| 29 | Butyrolactone | 18.42 | 1694 | na | na | — | 81 | na | 0.25 | 0.21 | 0.00 | 0.58 | 0.20 |
| 30 | Unknown | 19.00 | 1707 | na | na | — | na | na | 0.11 | 0.31 | 0.00 | 0.19 | 0.20 |
| 31 | Unknown | 19.65 | 1721 | na | na | na | na | na | 0.25 | 0.32 | 0.26 | 0.44 | 0.29 |
| 32 | Unknown | 20.36 | 1736 | na | na | na | na | na | 0.23 | 0.18 | 0.26 | 0.18 | 0.11 |
| 33 | Naphthalene | 20.59 | 1741 | 91 | 91 | 91 | 90 | 91 | 6.71 | 7.83 | 7.48 | 9.26 | 4.06 |
| 34 | Azulene | 20.66 | 1742 | — | — | — | — | 91 | 0.00 | 0.00 | 0.00 | 0.00 | 2.68 |
| 35 | Unknown | 20.73 | 1744 | na | na | — | — | na | 1.49 | 0.08 | 0.00 | 0.00 | 0.47 |
| 36 | Oxime-, methoxy-phenyl- [= Phenylacetaldehyde oxime] | 22.25 | 1773 | 86 | 91 | 86 | 90 | 91 | 0.60 | 1.47 | 1.05 | 1.83 | 0.99 |
| 37 | Ethanol, 2-(2-ethoxyethoxy)- [= ethyl carbitol] | 22.60 | 1780 | 90 | 90 | 91 | 91 | 90 | 5.56 | 1.74 | 1.79 | 1.40 | 2.66 |
| 38 | Cyclotrisiloxane, hexamethyl- | 23.23 | 1792 | 86 | 86 | na | na | na | 0.18 | 0.55 | 0.49 | 0.68 | 0.29 |
| 39 | Unknown | 23.72 | 1801 | na | na | na | — | — | 0.28 | 0.04 | 0.15 | 0.00 | 0.00 |

|  |  |  |  | Identity likelihood | | | | | Percentage of total peak area | | | | |
| --- | --- | --- | --- | --- | --- | --- | --- | --- | --- | --- | --- | --- | --- |
| No. | Compound name ^a^ | Mean RT (min) | Kovats Index | Hermaphroditic flowers | | | Staminate flowers | | Hermaphroditic flowers | | | Staminate flowers | |
|  |  |  |  | II | III | IV | II | V | II | III | IV | II | V |
| 40 | 2-Oxabicyclo[2.2.2]octan-6-ol, 1,3,3-trimethyl- | 23.83 | 1802 | — | 97 | — | na | 94 | 0.00 | 0.69 | 0.00 | 0.83 | 1.31 |
| 41 | Unknown | 23.89 | 1804 | na | na | na | — | na | 0.29 | 0.10 | 0.62 | 0.00 | 0.06 |
| 42 | Benzyl alcohol [= Benzaldehyde] | 24.10 | 1807 | 97 | 97 | 97 | 97 | 98 | 0.91 | 0.79 | 1.11 | 1.04 | 0.88 |
| 43 | Benzothiazole | 25.44 | 1830 | 94 | 94 | 94 | 94 | 94 | 4.55 | 2.46 | 3.52 | 3.00 | 2.75 |
| 44 | Benzene, 2-methoxy-4-methyl-1-(1-methylethyl)- [= Thymol methylether] | 26.06 | 1840 | — | 86 | 86 | 80 | 80 | 0.00 | 1.03 | 7.07 | 1.60 | 0.95 |
| 45 | Unknown | 26.13 | 1841 | na | na | — | — | na | 0.71 | 0.04 | 0.00 | 0.00 | 0.14 |
| 46 | 4-Acetylbenzoic acid | 26.17 | 1842 | na | 80 | — | — | na | 0.46 | 0.67 | 0.00 | 0.00 | 0.19 |
| 47 | Ethanol, 2,2'-oxybis- [=Diethylene glycol] | 26.61 | 1849 | 83 | 83 | 83 | 83 | 83 | 6.25 | 14.76 | 14.22 | 5.40 | 16.87 |
| 48 | Octanoic Acid [= caprylic acid] | 28.05 | 1871 | 87 | 86 | na | 86 | 96 | 1.53 | 0.88 | 0.65 | 0.76 | 0.98 |
| 49 | Benzoic acid, 4-ethoxy-, ethyl ester | 29.72 | 1896 | 96 | 94 | 97 | 96 | 97 | 1.27 | 0.71 | 0.64 | 1.17 | 0.63 |
| 50 | Nonanoic acid [= pelargonic acid] | 29.99 | 1900 | 93 | 94 | na | 93 | 92 | 1.35 | 0.77 | 0.15 | 0.50 | 1.12 |
| 51 | Caprolactam | 30.71 | 1910 | 96 | 96 | 90 | 94 | 95 | 1.58 | 0.27 | 0.53 | 0.27 | 0.57 |
| 52 | Benzene, 1,2,3-trimethoxy-5-(2-propenyl)- | 30.92 | 1912 | — | — | 98 | — | — | 0.00 | 0.00 | 0.29 | 0.00 | 0.00 |
| 53 | Diethyltoluamide | 32.16 | 1929 | 91 | 95 | 91 | 93 | 95 | 3.96 | 0.11 | 0.34 | 0.00 | 0.91 |
| 54 | Phenol, 2,4-bis(1,1-dimethylethyl) | 32.29 | 1931 | 93 | 94 | 93 | — | 91 | 1.91 | 1.24 | 1.32 | 1.77 | 1.42 |
| 55 | Unknown | 35.82 | 1975 | na | na | na | na | na | 0.23 | 0.22 | 0.26 | 0.24 | 0.21 |
| 56 | Unknown | 36.54 | 1983 | na | na | na | na | na | 0.89 | 0.79 | 1.80 | 0.86 | 0.64 |
| 57 | Unknown | 37.36 | 1992 | na | na | — | na | na | 0.95 | 0.38 | 0.00 | 0.18 | 0.48 |
| 58 | 7,9-Di-tert-butyl-1-oxaspiro(4,5)deca-6,9-diene-2,8-dione | 39.15 | 2012 | — | na | 98 | — | — | 0.00 | 0.04 | 0.45 | 0.00 | 0.00 |
| 59 | 1,4-Benzenediol, 2,5-bis(1,1-dimethylethyl)- | 41.01 | 2032 | na | 81 | na | 91 | 81 | 1.42 | 1.13 | 1.08 | 1.36 | 1.28 |
| 60 | 1,4,7,10,13,16-Hexaoxacyclooctadecane | 44.23 | 2064 | 93 | 80 | na | — | 81 | 2.70 | 0.06 | 2.16 | 0.00 | 0.34 |
| 61 | Unknown | 48.71 | 2104 | na | na | — | — | na | 0.29 | 0.10 | 0.00 | 0.00 | 0.04 |

Note. Compounds arranged according to retention time, excluding those identified only from blank controls and those with < 0.25% of peak area. Compounds with identity likelihood estimated < 80% listed as ‘Unknown.’ na = not available.

^a^ Common names in brackets, if applicable.
